# Impact of Predicting Health Care Utilization Via Web Search Behavior: A Data-Driven Analysis

**DOI:** 10.2196/jmir.6240

**Published:** 2016-09-21

**Authors:** Vibhu Agarwal, Liangliang Zhang, Josh Zhu, Shiyuan Fang, Tim Cheng, Chloe Hong, Nigam H Shah

**Affiliations:** ^1^Biomedical Informatics Training ProgramStanford UniversityStanford, CAUnited States; ^2^Baidu IncSunnyvale, CAUnited States; ^3^Stanford UniversityDepartment of Electrical EngineeringStanford UniversityStanford, CAUnited States; ^4^Center for Biomedical Informatics ResearchStanford UniversityStanford, CAUnited States

**Keywords:** search behavior, geotagged search logs, health care utilization, utility, health care costs, Internet

## Abstract

**Background:**

By recent estimates, the steady rise in health care costs has deprived more than 45 million Americans of health care services and has encouraged health care providers to better understand the key drivers of health care utilization from a population health management perspective. Prior studies suggest the feasibility of mining population-level patterns of health care resource utilization from observational analysis of Internet search logs; however, the utility of the endeavor to the various stakeholders in a health ecosystem remains unclear.

**Objective:**

The aim was to carry out a closed-loop evaluation of the utility of health care use predictions using the conversion rates of advertisements that were displayed to the predicted future utilizers as a surrogate. The statistical models to predict the probability of user’s future visit to a medical facility were built using effective predictors of health care resource utilization, extracted from a deidentified dataset of geotagged mobile Internet search logs representing searches made by users of the Baidu search engine between March 2015 and May 2015.

**Methods:**

We inferred presence within the geofence of a medical facility from location and duration information from users’ search logs and putatively assigned medical facility visit labels to qualifying search logs. We constructed a matrix of general, semantic, and location-based features from search logs of users that had 42 or more search days preceding a medical facility visit as well as from search logs of users that had no medical visits and trained statistical learners for predicting future medical visits. We then carried out a closed-loop evaluation of the utility of health care use predictions using the show conversion rates of advertisements displayed to the predicted future utilizers. In the context of behaviorally targeted advertising, wherein health care providers are interested in minimizing their cost per conversion, the association between show conversion rate and predicted utilization score, served as a surrogate measure of the model’s utility.

**Results:**

We obtained the highest area under the curve (0.796) in medical visit prediction with our random forests model and daywise features. Ablating feature categories one at a time showed that the model performance worsened the most when location features were dropped. An online evaluation in which advertisements were served to users who had a high predicted probability of a future medical visit showed a 3.96% increase in the show conversion rate.

**Conclusions:**

Results from our experiments done in a research setting suggest that it is possible to accurately predict future patient visits from geotagged mobile search logs. Results from the offline and online experiments on the utility of health utilization predictions suggest that such prediction can have utility for health care providers.

## Introduction

Over the past years, Internet search engines have changed the way people report health outcomes and/or seek information regarding symptoms, diseases, and treatments, resulting in a parallel growth of a large amount of medical information. The potential of addressing public health challenges and advancing medical research through the analysis of such information repositories, as well as the challenges inherent in working with such sources, are being recognized [1-4]. In a novel study, the use of click-through statistics generated by a commercial Web advertising service was shown to be an effective strategy for influenza surveillance [5]. As a Web-scale repository of patient-generated information, Internet search logs have been mined for diverse applications, such as screening patients with pancreatic adenocarcinoma [6] and discovering adverse drug events [7,8]. The use of Internet searches as an indicator of individuals’ interests and concerns related to health care has been studied for understanding the relationship between health anxiety and its effect on information-seeking behavior [9]. Notably, through the analysis of search logs collected from consenting users via a browser toolbar and complementary surveys, it has been shown that the analysis of long-term search behavior reveals patterns that may serve as markers for a transition to health care utilization [3].

Viewed against the backdrop of the recent tectonic shifts in the health care landscape in the Unites States, search log repositories present an opportunity to understand the nature of interactions between health care organizations and users—specifically interactions that result in utilization of health care resources. Gaining such an understanding is crucial for improving efficiencies and eventually, the accessibility of health care services [10,11]. Because search log data closely mirrors users’ daily concerns and activities, they contain embedded clues about imminent health episodes. As a result, predictions of health care utilization based on geotagged search histories provide a snapshot of future health care demand that is based on an aggregation of personalized health trajectories.

Logs of Internet searches initiated from mobile devices contain search text and time stamp information, as well as the location of where the search was initiated. The location information in search logs contains clues about the searcher’s interactions with the real world. For instance, consecutive searches from approximately the same location that are separated by a significant span of time could indicate an engagement at the particular location. The information utility of an individual’s approximate location within a virtual geographical boundary (referred to as a “geofence”) has been studied extensively within the ubiquitous computing community and forms the basis of several location-based services [7,9,12].

Based on deidentified searches and distances of searches from medical facilities, White and Horvitz [2] have argued that the evidence of health care utilization is related to the acuity of symptom searches. Although such observational analysis of Internet search logs has shown the feasibility of the approach for discovering population-level patterns, the utility of the endeavor to the various stakeholders in a health ecosystem remains unclear. Among other things, assessment of the utility of a predicted health outcome depends on the cost of making the prediction, the “actionability” of the prediction [13], as well as individual goals and value perception [14]. In general, the determinants of predictive utility are hard to measure and possibly subjective. As of this writing, we are not aware of a study that evaluates data mining experiments on Internet search logs from a utility standpoint.

**Figure 1:**
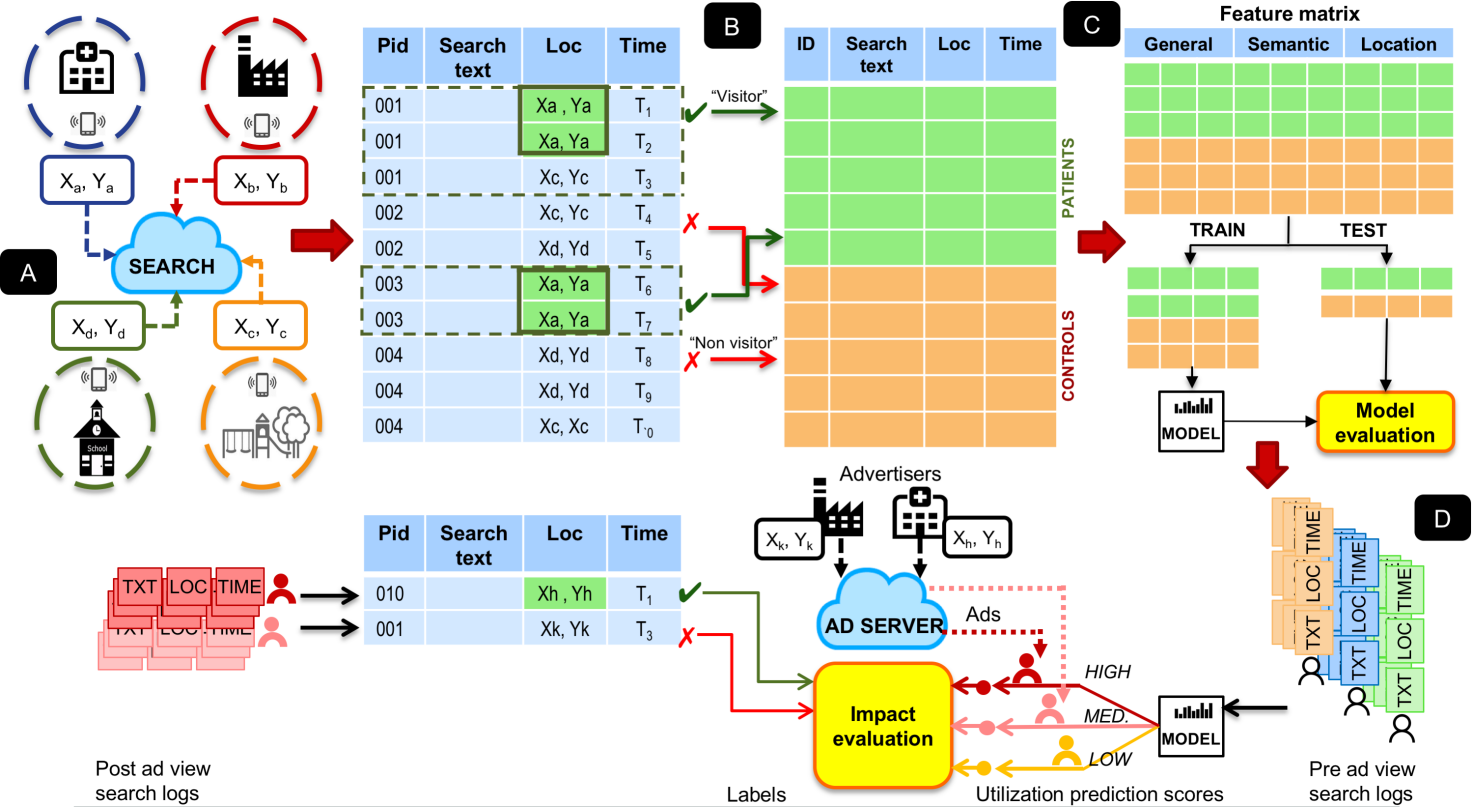
Overall study design: (A) generation of search logs based on searches within geofences, (B) identification of searches proximal to medical facilities and selection of patients and controls based on filtering criterion, (C) learning statistical model for predicting health, (D) evaluation of impact on ad show conversion rates for search users with high utilization prediction scores prior to ad views.

In this study, we assess the utility of predicting health care utilization from the health care provider’s perspective in the context of behaviorally targeted advertising. Recent studies on Internet consumer behavior have sought to model the publisher’s and the advertiser’s payoffs in terms of the performance metrics of a behaviorally targeted advertising campaign [15,16]. The advantage of using such a framework is that it allows for a fine level of control on experimental parameters and produces a concrete measure of the impact of health care utilization predictions within a specific setting. We compute features representing different aspects of search behavior along with surrogate measures of health care resource use directly from users’ search logs and train statistical models to predict future health care utilization from search logs. Our models incorporate features that summarize temporal trends in the semantic and location patterns of searches and allow us to investigate the differential effect of various classes of features on utilization prediction. We then evaluate the impact on ad show conversion rates for search users who were shown ads and whose utilization prediction scores were computed from historical search logs. Our overall study design is depicted in Figure 1.

## Methods

### Data

Our dataset consisted of deidentified mobile Internet search logs representing more than 1 billion searches from 9.5 million search users of the Baidu search engine from March 2015 to May 2015, made accessible to the authors under a collaborative research program. The search logs contain search text (Chinese), the time stamp, and the location (latitude and longitude) of the search, and represent searches made from locations within China. Identifying health care utilization based on evidence of searches on mobile devices that were made close to hospitals is prone to false positives and negatives. Search users may work inside or close to hospital locations or may be passing by one, and may not be consumers of health care resources at the time they make the search from locations proximal to a hospital. Similarly, search users may visit a hospital as patients but not search during their visit. We acknowledge that it is not possible to completely eliminate false positives and false negatives when labels are assigned purely on the basis of searches made within the geofences of medical facilities. However, we have successfully reduced the number of false positives in our data by explicitly filtering out “weak” labels.

### Inclusion and Exclusion Criteria

From all searches that took place between the aforementioned dates, we discarded search users who searched from within 200 meters of a hospital but the evidence of their presence in that location was less than 900 seconds. We also discarded search users who had more than 15 searches in a month from locations in the proximity of a medical facility because these may be individuals who lived or worked close by, or they may be health care professionals. Finally, we excluded users who searched more than five times in a month in the vicinity of different medical facilities. Search logs for the remaining 4 million search users were deemed to have been evidence of visiting a medical facility and 1.5 million distinct search users were randomly sampled from them. We drew a proportionate random sample from all searches, which did not originate within the geofence of a known medical facility, to obtain 8 million distinct search users with no evidence of visiting a medical facility, thus giving a total set of 9.5 million search users. In the absence of relevant user information, we matched our controls on the number of days of available search logs.

Because we were interested in studying the temporal characteristics of search logs that culminated in the visit to a medical facility, we selected those who had search logs for each of 42 or more days preceding their last visit to a medical facility (a higher threshold, we discovered, would reduce our cohort size significantly and adversely impact statistical power). In the remainder of our paper, we refer to this cohort as “patients.”

### Longitudinal Partitioning

**Figure 2:**
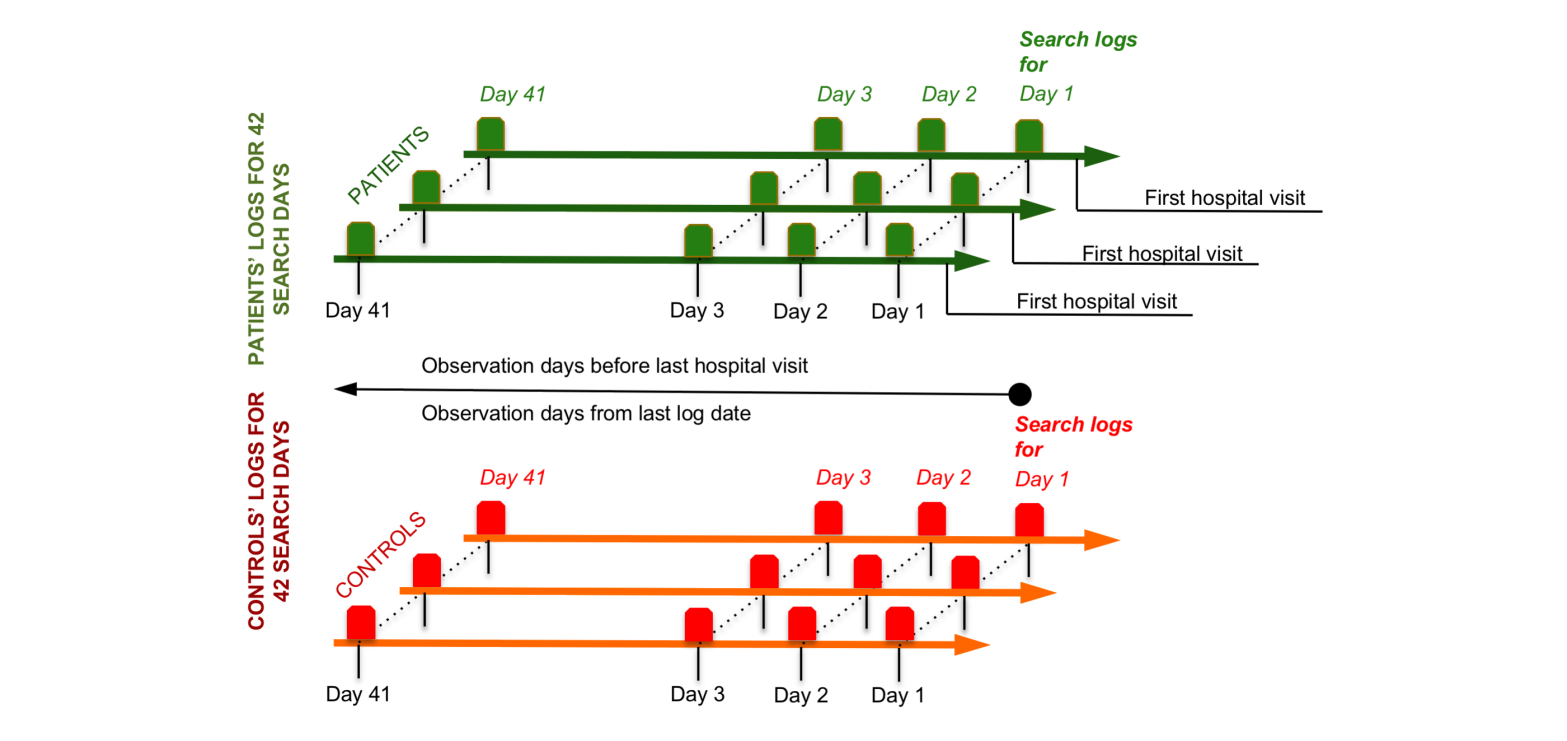
Longitudinal partitioning of search log data with analysis windows covering 41 search days. Control end points were selected randomly.

We partitioned the search logs for patients and controls by search days, in which search day *n* is the *n* th day in the sequence of days for which logs are available for a search user, preceding an endpoint. For patients, we defined the endpoint as the date of their first visit to a medical facility. For controls, the endpoint was picked randomly from each user’s search log. After excluding the last day of visiting a medical facility, we could define an analysis window comprising 41 consecutive search days for patients. We defined a similar analysis window with 41 consecutive search days for controls with an endpoint coterminus with the first search day. Figure 2 illustrates the longitudinal partitioning of our search log data as previously described.

### Feature Engineering

We chose three classes of features to study the discriminatory patterns in search logs across patients and controls over a succession of search days. The classes, as described in Table 1, represent general attributes of search logs, semantic properties of the search text, and the location attributes of search logs for each search day in the analysis window. We also created aggregated features that were based on counts of search log properties aggregated over the entire analysis window.

**Table 1:** Description of feature categories.

Feature categories and description		Aggregate	Daywise
**General**			
	Number of searches	Yes	Yes
	Number of health care-related searches	Yes	Yes
	Mean session duration	Yes	Yes
	Mean length of search text	Yes	Yes
	Session interval reduction score	No	Yes
**Semantic**			
	Number of searches for a disease	Yes	Yes
	Number of searches for a drug	Yes	Yes
	Number of searches for a medical device	Yes	Yes
	Number of searches for a medical procedure	Yes	Yes
	Number of searches containing one of 100 enriched (Chinese) words	Yes	Yes
**Location**			
	Number of searches mapped to one of 53 enriched location categories	Yes	Yes
	Number of searches whose location labels contain one of 113 words	Yes	No

### General Features

It has been shown that the linguistic structure of Internet searches influences information retrieval from Web search engines [17]. In a study comparing search characteristics that originate from mobile devices to searches that originate from PCs, Jadhav et al [18] showed that health search queries tend to be longer than general search queries. We chose to include attributes related to the length of the text search and the duration of a search session among our general features. A search session represents several searches linked together by the most prominent themes returned in search results and longer sessions are likely to suggest the evolution of a search user’s interest from general to more specific concepts. We also included the number of searches and number of health care-related searches in both our aggregate and daywise features because a user’s level of concern about a health care issue is likely correlated to the number of times they search for information for reassurance or remedy.

### Interval Reduction Score

**Figure 3:**
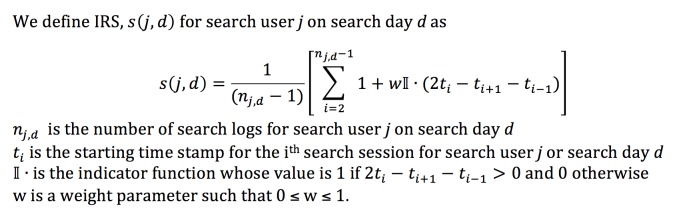
Equation for the Interval Reduction Score (IRS).

Earlier studies on health information-seeking behavior of Internet users have studied the relationship between search behavior and health concerns of search users. For instance, it has been shown that physician information-seeking behavior, as assessed by the mean page view duration for specific websites, is distinct from general online media activity [19]. Health anxiety in patients has also been shown to be associated with specific search patterns, such as intense search activity punctuated by periods of calm [20]. With the intention of capturing differences in such patterns between utilizers and nonutilizers within our data, we computed an interval reduction score (please see Multimedia Appendix 1) for each search day for each user (Figure 3).

For search days with two or fewer searches, the interval reduction score was 1. We experimented with different values of w and obtained best results with small values close to 0.1 and by only considering health care-related searches in a search day.

### Semantic Features

The language of the search queries in our dataset posed a unique challenge to our analysis. On one hand, we could leverage advantageous aspects of the Chinese language, such as the lack of verb conjugation and plural forms. In addition, this allowed us to capture the meaning behind idiomatic expressions that could be challenging to translate. On the other hand, English tokens would enable us to use a wider variety of existing language analytic tools. Thus, we balanced the two approaches by analyzing our tokens in Chinese, and performing token translation and carrying out further analysis in English.

**Figure 4:**
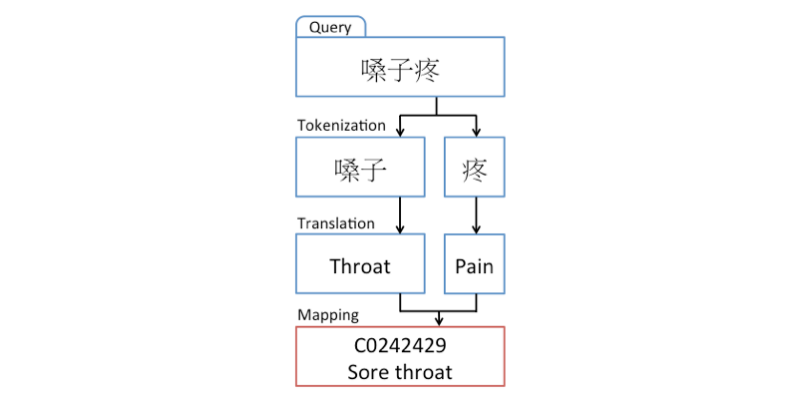
Framework for search text translation and mapping.

For our Chinese semantic analysis, we identified enriched tokens that were used by patients and controls using a Fisher test with Bonferroni correction. We also evaluated the number of patients and controls that searched for each token on any given day, and compared the term frequency between patients and controls. We took the union of the tokens from these two analyses, and the best performing 100 tokens were included as features in subsequent analysis, after manually inspecting the features for procedure artifacts. Following this analysis, all tokens from health care queries were translated from Chinese to English for downstream analyses (Figure 4).

To model the variation in search content across search days for patients and controls, we further chose to explicitly characterize the medical content within the search text by using an approach that has been validated in clinical text mining research.

Although the form and structure of search text is fundamentally different from the free text in patients’ records, we noted that certain aspects of the two were strikingly similar from a linguistic standpoint. Intuitively, one may take advantage of this similarity by employing proven tools and techniques that have been used to characterize lexical coverage in the former for achieving similar goals with the latter. In particular, the use of ill-formed sentences, abbreviations, and spelling errors are common to both search text and clinical text, and motivated our choice of a biomedical terminology for identifying and delineating the use of medical terms in search text. We decided to use an extensive terminology of terms drawn from 22 clinically relevant ontologies from the Unified Medical Language System (UMLS) and BioPortal [21]. The lexicon represents more than 3.1 million terms that map to nearly 1.2 million concepts and a functional evaluation of annotations of clinical text based on the same have shown equivalence with more sophisticated natural language processing-based approaches [22]. Because the UMLS provides a mapping from each concept to one or more semantic types, by coalescing relevant semantic types into groups that represent diseases, drugs, devices, or procedures, one may achieve a fine-grained characterization of search terms by determining their group membership. However, because the semantic types as defined by the UMLS semantic network represent key relationships between biomedical concepts, inferring the medical semantics of search text using these semantic types is likely to be noisy. Results from our initial experiments showed many examples of misattributions on account of the domain specificity of our lexicon. For example, commonly occurring terms in search texts (eg, dame, gift, blade) are mapped to medical concepts that are grouped as a drug (D-Ala2-methionine enkephalinamide), a procedure (gamete in fallopian transfer), and a device, respectively. To address this issue, we investigated whether such misattributions were more pronounced for certain semantic groups than others. We segregated search texts in our training set into health care and non-health care categories on the basis of whether a token in the translated text mapped to any one of the four high-level semantic groups mentioned previously. We then compared the segregation with an independent categorization based on Baidu’s knowledge graph. We then repeated the experiment, each time leaving out one of the four semantic groups for making the health care categorization of search texts.

We observed that leaving out the device subgroup results in an overall improved agreement between the two segregations (from 15.2% with all four groups to 40% when only using drugs, diseases, and procedures), whereas leaving out other groups did not show improvement. Therefore, we used membership counts of individual searches into the groups drugs, diseases, and procedures for features indicating the nature of the medical content in queries on a given search day. We noted that the semantic groups for drugs, diseases, and procedures represented the largest grouping of concepts within the UMLS [23-25] and, hence, are likely to be the most relevant groups in a lexicon-based semantic analysis approach. Similarly, membership counts across the full analysis window yielded the aggregated semantic features related to medical content.

### Search Specificity

Information returned by medical searches is known to influence concerns related to health, which in turn modulates subsequent search behavior [20]. Concerns about common symptomatology have been shown to escalate into searches for serious and rare diseases [26] and anxiety regarding one’s health is likely to influence health care utilization intent [3], possibly precipitating a visit to a medical facility. Thus, we were interested in modeling the evolution of searches that progressed from a general inquiry regarding symptoms into a specific inquiry regarding a serious health condition. We chose to use the information content score of the most specific term in a search as an indicator of the generality of the subject matter of the search. The information content score takes advantage of the hierarchical structure of a medical ontology to ensure a monotonically nondecreasing measure of specificity and may be computed based on document-level frequency of a term in a corpus. For search terms that mapped to our medical terminology, we computed session-level information content scores from the segmented search texts. As with other semantic features, we chose the highest daywise score for all searches from a given search day for a daywise measure of search specificity.

### Location Features

To build location features, we attached location labels to the latitude and longitude coordinates of searches. We used the Gecko Landmarks (Gecko Landmarks Ltd, Espoo, Finland) application program interface (API), which takes the latitude and longitude as input and outputs the 10 closest landmarks to this referenced location ranked by distance along with name and category labels for each landmark. For example, for a reference location given by latitude 39.903651 E, 116.415505 N, the Gecko API returns Beijing Hospital as the closest landmark with a category label of “hospital.”

**Figure 5:**
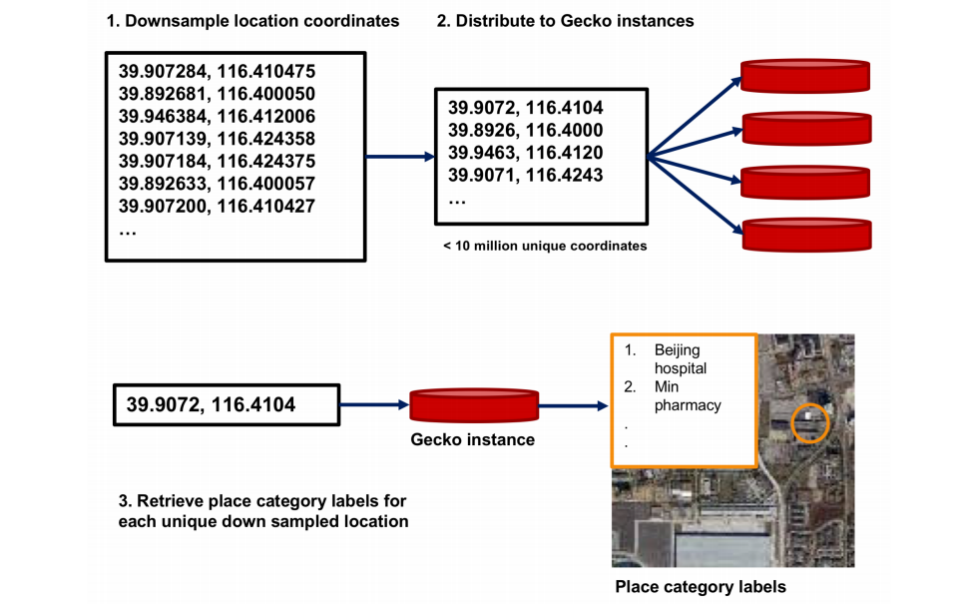
Extraction of place category labels.

We rounded each geographic coordinate to four decimal places and then filtered for uniqueness. This resulted in less than 10 million coordinate pairs without a significant loss of precision. Coordinates with accuracy up to four decimal places represented an accuracy of approximately 11 meters, which we considered adequate for our location features. We then obtained access to a rate-limited instance of a Gecko server and carried out a batchwise conversion of our unique, binned coordinates to obtain the respective location labels (Figure 5). The batchwise lists of landmarks were merged and mapped back to the original location coordinates from which we built our feature matrix. Specifically, the features were the number of searches made by a user from a given landmark category (eg, health, restaurant). We also created features based on individual words in the location names. For example, searches from an educational building could have the words “elementary” or “university” in the location names that were identified by the Gecko API. Although both universities and elementary schools have a category label of “education,” they have opposite effects on predicting hospitalization; the former term is enriched whereas the latter is slightly depleted in patient’s search logs. We indicated the presence or absence of a word token in a search location name using a binary (0,1) variable. Features based on individual tokens in location names captured additional granularity without imposing a structure on the location names a priori.

### Building Prediction Models

We constructed a variety of supervised machine-learning models based on our aggregate and daywise features. In fitting our models, the aggregate feature set and the daywise feature sets were divided into 80% for training and the remaining 20% for testing. Given the sparsity and correlations within our features, we focused primarily on using regularized models to reduce the dimensionality of our feature set and to avoid overfitting. All machine-learning analyses were performed using R 3.2.0 (R Development Core Team, Vienna, Austria). We selected linear, nonparametric, and ensemble methods to evaluate the best fit to our data. For linear models, we built lasso, ridge, and elastic net models using the “glmnet” package. Five-fold cross-validation was applied to the training set to determine the optimal tuning parameter lambda for lasso and ridge classification. For elastic net, a grid search was performed to determine lambda and alpha. The lambda that was within 1 standard error of the lambda that produced the minimum cross-validation error was selected for these models to prevent overfitting. In addition to linear models, we built support vector machine (SVM) models with a Gaussian kernel (using e1071) and random forest models (using the “randomForest” package). For our SVM models, gamma was set to 1 divided by the number of features, and the cost was chosen via cross-validation. To evaluate the performance of our models, we constructed receiver operating characteristic (ROC) curves. We used the area under the curve (AUC) of the ROC curve to compare the performance of our classifiers in the held-out test sets.

### Choosing the Most Informative Features

As described in the section on feature engineering, our initial feature design choices were guided by insights generated in prior work as well as by our experience of mining medical content. Our three feature categories attempt to discriminate between utilizers and nonutilizers based general search use, search contents, and search locations, respectively. Learning spatial trajectory patterns from location tags and learning linguistic patterns from embedded search texts requires the use methods from different subfields within machine learning, each an area of active research by itself. To guide further work on feature design and improvement, we measured the individual contribution of each of three feature categories on prediction performance. We trained three models on three different feature matrices, each containing features from only two of the three feature categories. Each model was tested against the held-out test data, featured in the same way.

### Measuring the Impact of Utilization Prediction

We validated our prediction model through an experiment conducted in Baidu’s mobile search ads system, which charges advertisers based on user clicks. The model used in online evaluation is modified to comply with commercial limitations (such as the use of location APIs). However, the model contains similar category of features, reconstructed from the raw search log data. Medical facilities that advertise via the system are interested in a low cost per conversion (CPC) and a higher show conversion rate, which implies more efficient use of the advertising budget. Our objective was to measure the relationship between ad conversions and the prediction of health care utilization. In particular, we wished to evaluate if displaying medical facility advertisements to predicted health care utilizers results in higher show conversion rate, “conversion” being defined as a single health care utilization by a search user, satisfying the following two conditions: (1) the search user had no utilization of the same medical facility within 1 month before the conversion, and (2) the utilization took place within 2 weeks after the search user was shown the ads from this specific hospital.

Intuitively, the first condition restricts the conversion to be a new hospital utilization rather than a readmission, while the second encourages a relationship between ads display and hospitalization. A rigorous argument establishing the linkage between condition 2 and causality is beyond the scope of this paper. Instead, we refer the interested reader to relevant work in this area [27,28] and the references therein.

### Offline Evaluation

**Figure 6:**
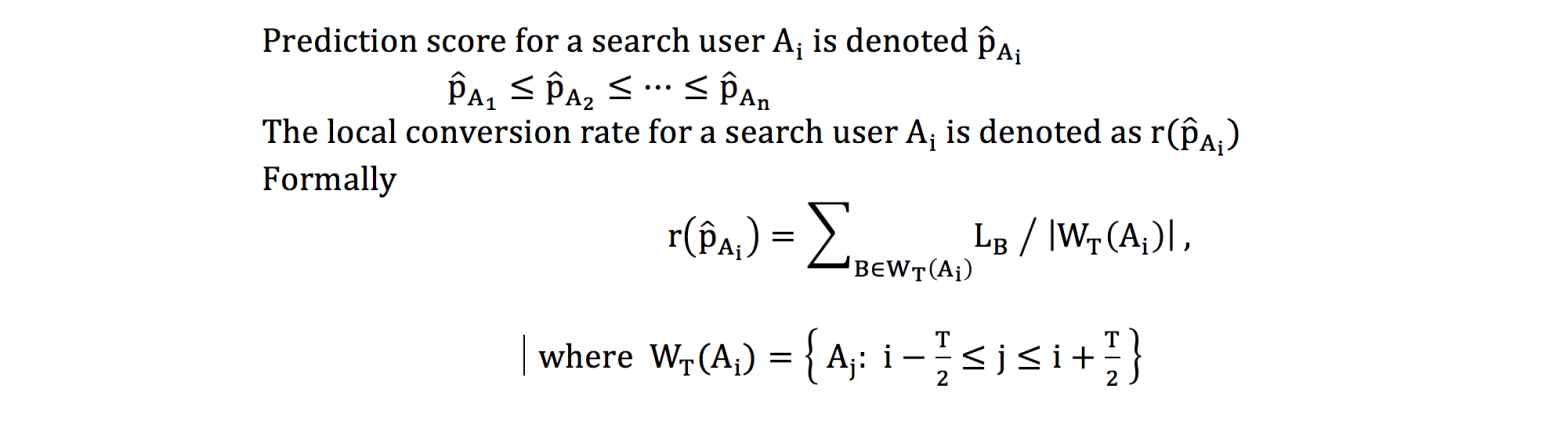
Equation for local show conversion rate.

We carried out an offline evaluation prior to deploying the model in a production setting. Our goal was to ascertain the efficacy of the predicted health care utilization scores on real posterior conversion data. We first collected logs of all search users who had been shown medical facility advertisements between June 1 and June 7, 2015. Using our prediction model, we generated health care utilization scores for these users based on their search logs from May 2 through May 31, 2015. For each search user A, we obtained a predicted health care score (a real number between 0 and 1) and a conversion label (0 or 1). We adopted the following metric, which is considered to be more interpretable for the evaluation task at hand compared to the conventional AUC. We ranked these search users by their predicted scores and plotted the show conversion rate versus risk score. The local show conversion rate for a search user is defined as the conversion proportion within a T-person window centered at the risk score for the user (Figure 6). This local show conversion rate at a given risk score represents the approximate conversion probability of people with scores close to the given risk score and is aligned with the way our model was adopted in the online experiment (described in the following sections).

### Online Evaluation

Health care utilization prediction scores were generated on a daily basis from search users’ previous day’s logs. Feature generation and prediction was carried out using Baidu’s MapReduce framework. MapReduce jobs running on a cluster with 50 HPC machines, composed of 550 physical cores and 6.4 terabytes of memory in total typically take 3 hours for feature and score generation. The predicted scores are stored in Baidu’s online k-v servers with 70 gigabytes of memory, where the online ads system can use them to modify the ads bidding coefficients. The online evaluation was carried out on approximately 10 million mobile users of Baidu apps. The user pool for this experiment is large enough to avoid user-to-user variations. We split these users into two parts to conduct the A/B test. For each user in the treatment group B, the predicted score was mapped to a coefficient in the interval (0.7, 1.3), which was then used to adjust the probability that a health care ad was displayed, whereas the control group A was left unchanged.

## Results

### Predictive Model for Health Utilization

The AUCs for our feature sets across all models are shown in Table 2. Our models showed the poorest performance with the aggregate features, with the highest AUC at 0.627 from the lasso model. On the daywise features, we observed the best performance with random forest, reaching an AUC of 0.796. The AUC when a single feature category was omitted was 0.781, 0.789, and 0.779 when the omitted categories were semantic, general, and location, respectively. Omission of location features caused the largest drop in AUC, followed by the omission of semantic and general features. We used a random forests classifier with daywise features for measuring the individual contribution of the feature categories.

**Table 2:** Areas under the curve for aggregate and daywise feature sets.

Model	Aggregate		Daywise	
	training	Test	training	Test
Lasso	0.899	0.627	0.899	0.589
Ridge	0.890	0.598	0.920	0.639
Elastic net	0.901	0.601	0.920	0.621
Support vector machine, radial kernel	0.983	0.590	—	—

### Predicting Utilization by Specialty

Many of the geofenced locations allow sublocalization of searches via network access points that served as sublocation indicators. For medical facilities where this was feasible, the user search logs could be tagged with access point identifiers that were, in turn, mapped to hospital departments. We collapsed department tags into four broad categories covering treatments related to male utilizers, treatments related to female utilizers, beauty treatments, and other specialty treatments, and investigated the feasibility of learning search log predictors that may classify a health care utilization by visit type. For a random forests classifier trained on our daywise features, we obtained a held-out test accuracy of 0.632 and AUC of 0.592, where AUC for the multiclass classification was computed as a mean of all pairwise comparisons as discussed by Hand and Till [29].

### Feature Importance

An examination of the top 30 positive and negative features from our daywise models (tabulated in Multimedia Appendix 2) indicates a high degree of overlap among the top ranking features. The overlap increased if we disregarded the day for which the feature was selected. All features pertained to search log events within 15 search days preceding the outcome (health care utilization). The number of search sessions per search day was among the top features for all models except the elastic net. The interval reduction score feature, which summarizes the lengthening/shortening of search intervals for all searches made by a user per day, appeared among the top 30 positively associated features in two of our models. The number of visits to place categories labeled as accommodation or health care facility appeared as important features in three of the daywise models.

### Offline Evaluation

**Figure 7:**
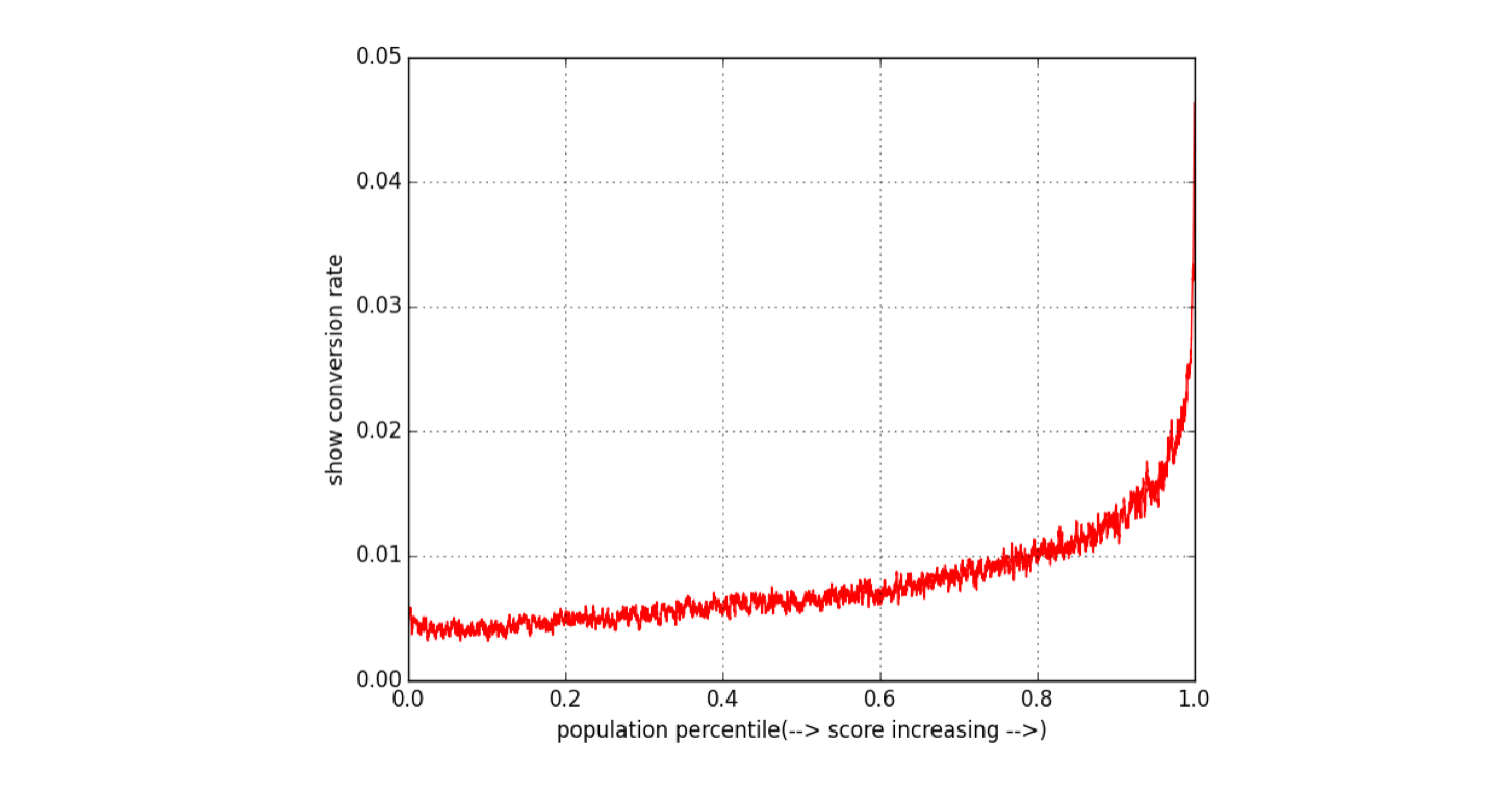
Show conversion rate versus utilization prediction score.

Figure 7 shows the expected change in the show conversion rate with changes in the prediction risk score. Note that the horizontal axis has been normalized and represents the percentile of population with increasing predicted scores. Therefore, the AUC approximates the proportion of all converted ad views. Our offline experiment using data from May 2 through May 31, 2015, resulted in a monotonically increasing curve confirming that a high predicted score implies a high show conversion rate; thus, justifying our online experiment.

### Online Evaluation

Several metrics including show conversion (the ratio between number of ads shown and hospital visits) and the cost per action (CPA) for shown conversions were tracked in the online experiment for the time period between August 24 and September 27, 2015. The reported numbers are the relative change between the experiment group and the control group. For reasons of confidentiality, we do not disclose the revenue of the advertisers. Two models were applied separately on two experiment groups, one had location-based features and the other did not. As expected, the model with location features showed a higher conversion (3.96% vs 0.67%) and lower CPA (–1.77% vs 1.61%).

## Discussion

The value of Internet search logs as valuable repositories of patient-generated biomedical information is widely appreciated. Because location data offer an opportunity to link search users’ virtual behavior with their real-world activities, it holds promise for many areas of medical research that have heretofore remained impregnable to conventional research techniques.

### Principal Results

The experiments described in our study demonstrate the relationship between predicted health care utilization and show conversion rate of targeted advertising (our surrogate measure of utility). The show conversion evaluates how many hospital visits happen after we show health care-related ads to the users and demonstrates that our online evaluation results are in good agreement with the model. Both models (with or without location-based features) have higher show conversion, suggesting users in experiment groups are prone to be affected by those advertisements. The model with location-based features has higher change of show conversion, which is consistent with results that the performance drops most suddenly when omitting the location features. The CPA is a similar metric to the CPC, which considers the cost per show conversions. It is observed that the location-based features helped lower CPA.

### Limitations

With its high sparsity, missingness, and vulnerability to contamination, geotagged search data also pose unique challenges for informatics research. A key limitation is the vulnerability to noise from searches close to the observation boundaries, as well as from false negatives that arise from our labeling approach. We expect that search data that span larger observation windows will allow creation of clear feature sets. Methods that attempt to learn from only positive labeled data may also be explored to control for false negative labels. Although feature sparsity in longitudinal patient histories is an active area of research in clinical informatics, search log texts add a new dimension to the challenge in at least two ways. First, given the “consumer” nature of the search texts, identifying biomedical content requires the use of a consumer health lexicon as well as session-level context detection, unlike patient notes where a term within the context of the note unambiguously represents a concept. Secondly, the “Internet scale” of the data and the approximated truth labels result in long-tailed distributions of potential health care concepts (eg, the occurrence of the token “gift” in health care utilizers’ searches versus in nonutilizers’ searches).

The relationship between the performance of prediction algorithms and the impact on utility—analogous to net reclassification in clinical studies [30]—has not been examined in our work. Specifically, the decision rules mapping a health care utilization prediction score to the probability of a health care advertisement being served impacts the overall show conversion rate. For example, a prediction model with a high AUC (capable of a high true positive rate and a low false positive rate simultaneously) coupled to a decision rule that results in advertisement displays to searcher users with low prediction scores would be inefficient. In general, this holds true in many health care prediction tasks where a high AUC may be a misleading indicator of the utility of the prediction model as it effectively leads to no net reclassification [30,31]. A full investigation into dynamic calibration of decision rules for mapping prediction scores to advertisement views and the impact on CPC metrics is beyond the scope of this study.

### Comparison With Prior Work

We note that mining of search texts for the purposes of syndromic surveillance has been actively studied in the past [32-34]. The study by Wang et al [35] in forecasting new outpatient visits related to dementia based on predictive tokens in search texts focuses on the problem of predicting health care utilization from the provider’s perspective. Nagar et al [36] have constructed spatiotemporal models based on tweets localized to New York for influenza surveillance. Our experiments suggest that in addition to using tokens as semantic predictors, features based on the location of the search improve the performance of utilization prediction models. Further, we were able to link click-throughs with subsequent geolocation search data to improvise a metric for assessing prediction impact.

Our work on predicting health care utilization from geotagged search logs is conceptually similar to the privacy-sensitive analysis of geotagged data from mobile devices by White and Horvitz [2] in that we make use of biomedical lexical resources to characterize the medical content in a search. However, our approach is novel in the use of a stack of temporal features based on a fixed analysis window of search days. The higher resolution of our approach is able to capture the progression of a wide variety of search attributes. Among our top daywise features, the number of queries on the search day preceding the day of a medical visit is selected by both the random forests and the ridge regression models and is in agreement with the results from White and Horvitz.

The association between health care utilization and temporal news trends has been examined previously [37]. A key finding in earlier studies on search log-characterized patient behavior is the escalation of health-related anxiety in the period leading to a health care utilization episode [8,21]. We believe that characterizing the temporal progression of relevant search features can reveal markers of anxiety escalation that precipitate health care utilization. In this work, we relied on the information content of search text tokens as a measure of the specificity of the text and compute the mean of the highest information content score (most specific token) of all searches made by a user within a search day. The mean information content score a few weeks prior to the hospital visit is selected by our L1 penalized logistic regression model as one of the top predictors. Given the sparse feature space (consisting of more than 1 million tokens), the L1 penalty results in the shrinkage of all but a small set of predictors. The mean information content score is unable to survive the selection criterion in models that do not have such sparsity-handling mechanisms.

In addition, we define another measure of anxiety—the interval reduction score. The interval reduction score, as defined in the Feature Engineering section, is the mean (daywise) shortening or lengthening of the interval between consecutive health care-related searches. For two of our daywise models, the interval reduction score in the 2 weeks prior to the hospital visit is among the top predictors. The models trained on daywise features for predicting health care utilization performed better compared to the models trained on aggregate features suggesting that daywise progression of search attributes better represents search behavior that signals health care resource utilization.

### Conclusions

Overall, the results of the two sets of experiments—first the proof-of-concept done in a research setting and second the offline as well as online experiments on the utility of health utilization predictions—support the claim that it is possible to accurately predict future patient visits from geotagged mobile search logs and that such prediction can have utility for health care providers.

## Multimedia Appendix 1

Interval Reduction Score.

## Multimedia Appendix 2

Top 30 daywise features.

## Abbreviations

API: application program interface
AUC: area under the curve
CPA: cost per action
CPC: cost per conversion
ROC: receiver operating characteristic
SVM: support vector machine
UMLS: Unified Medical Language System
