# Supplementary material for: Impact of Predicting Health Care Utilization Via Web Search Behavior: A Data-Driven Analysis
Source: J Med Internet Res. 2016 Sep 21;18(9):e251. doi: 10.2196/jmir.6240 (PMC5052461; doi:10.2196/jmir.6240)
Supplement: Supplementary file 1 [file jmir_v18i9e251_app1.pdf]

APPENDIX B

Interval Reduction Score

The interval reduction score for a search user and a given search day, represents the proportion of successively shortening inter-search intervals that occur within the searches for the given user and search day. Given time stamps for the ith and $(i+1)^{th}$ search session for search user *j* on search day *d* let,

$$p_i = t_i - t_{i-1}$$

The average number of successively shortening intervals on a day with $n_{j,d}$ healthcare searches is given by

$$\frac{1}{(n_{j,d}-1)} \sum_{i}^{n_{j,d}-1} k\, I \cdot (p_i > p_{i+1})$$

where k is a weight factor = 1 + w

The above is the same as

$$\frac{1}{(n_{j,d}-1)} \sum_{i}^{n_{j,d}-1} k\, I \cdot (2t_i - t_{i+1} - t_{i-1} > 0)$$
