# Supplementary material for: Impact of Predicting Health Care Utilization Via Web Search Behavior: A Data-Driven Analysis
Source: J Med Internet Res. 2016 Sep 21;18(9):e251. doi: 10.2196/jmir.6240 (PMC5052461; doi:10.2196/jmir.6240)
Supplement: Supplementary file 2 [file jmir_v18i9e251_app2.pdf]

APPENDIX A

Top 30 positive day-wise features	Top 30 negative day-wise features

	RFC	Ridge	L1	Eastic net		RFC	Ridge	L1	Eastic net
1	d7_□□	d4_□□	d1_□□	d8_□	1	d5_□	d11_□	d10_□	d9_□□
2	d6_□□	d3_□□	d3_□□	d1_□	2	d4_□	d4_two	d3_□	d4_□
3	d5_□□	d4_□	d9_six	d4_□	3	d3_□	d7_□	d15_□□	d12_two
4	d1_□□	d4_□	d2_□	d2_□□	4	d6_□	d13_□	d3_six	d11_□
5	d12_nurse	d6_□	d15_doctor	d4_homeVisit	5	d6_□	d6_□	d15_□	d9_□
6	d7_nurse	d1_□	d5_□	d9_□	6	d12_how	d4_□	d7_□□	d8_□
7	d8_□□	d3_□	d7_six	d3_□□	7	d5_□	d8_□□	d1_□	d4_□
8	d3_□□	d12_□	d2_homeVisit	d2_HE	8	d15_year2015	d6_two	d5_□	d3_□
9	d2_□□	d14_□	d2_□□	d14_□□	9	d15_□□	d6_□□	d9_□□	d11_□□□
10	d6_irs	d12_doctor	d11_□	d1_□□	10	d2_□	d1_□□	d10_□□	d11_year2015
11	d15_□	d7_□	d9_□	d14_□	11	d11_□	d11_□	d13_□	d10_□
12	d11_□	d12_six	d8_□	d7_□	12	d3_□	d3_□	d15_□□	d8_□□
13	d14_□	d15_□	d4_□□	d8_doctor	13	d11_□	d5_□	d6_□	d2_□
14	d12_□	d12_□	d13_□	d11_□□	14	d1_□	d6_□	d1_□	d8_□
15	d1_qid_count	d5_□	d1_□□	d14_□	15	d4_□	d11_□	d12_□	d11_□
16	d8_how	d8_□	d5_□□	d13_□	16	d15_□	d12_□	d6_□	d3_□
17	d2_three	d1_qid_count	d2_□□	d12_□	17	d10_□	d15_□	d2_one	d13_year2015
18	d13_□	d1_□	d1_□	d1_□	18	d1_□□	d9_□	d4_six	d9_□□
19	d6_AC_h	d15_□	d7_□	d15_□	19	d13_two	d3_□	d10_□□□	d2_year2014
20	d1_□	d2_□	d10_nurse	d3_□	20	d1_□	d13_□□	d5_□	d12_□
21	d15_□	d10_□	d9_□	d9_irs	21	d5_□	d14_□	d12_two	d9_□
	d1	d1	d6	d8		d12			d10

22	0_□	4_□	_□	_□	22	_□	d10_□	d5_two	_□
□ 23	□ d15_□	□ d13_□□	□ d12_□	□ d15_□□	□ 23	□ d15_□	□ d15_□	□ d6_□	□ d13_□□□
□ 24	□ d5_□	□ d5_□	□ d10_□	□ d11_□	□ 24	□ d11_two	□ d1_□	□ d15_two	□ d4_□
□ 25	□ d4_□□	□ d11_AC	□ d4_qid_count	□ d14_nurse	□ 25	□ d15_two	□ d17_□	□ d8_□	□ d6_□
□ 26	□ d1_doctor	□ d8_□	□ d2_□	□ d7_□□	□ 26	□ d13_□	□ d13_□	□ d2_□□	□ d12_□□
□ 27	□ d4_□	□ d3_□	□ d14_avgic	□ d15_□□	□ 27	□ d4_one	□ d14_□	□ d3_□□	□ d9_□
□ 28	□ d4_□	□ d11_□	□ d4_□	□ d2_□□	□ 28	□ d2_□	□ d11_□	□ d15_□	□ d2_□
□ 29	□ d12_□	□ d13_□	□ d6_□	□ d9_□	□ 29	□ d2_two	□ d12_how	□ d5_□	□ d1_□
□ 30	□ d12_□	□ ***d8***_□	□ d13_doctor	□ d13_□	□ 30	□ ***d15***_□	□ ***d10***_□□	□ d7_□	□ d11_□
